# Topical Anti-Inflammatory Effects of Quercetin Glycosides on Atopic Dermatitis-Like Lesions: Influence of the Glycone Type on Efficacy and Skin Absorption

**DOI:** 10.1007/s10753-025-02236-1

**Published:** 2025-01-14

**Authors:** Shih-Chun Yang, Zi-Yu Chang, Chien-Yu Hsiao, Abdullah Alshetaili, Shih-Hsuan Wei, Yu-Tai Hsiao, Jia-You Fang

**Affiliations:** 1 Department of Microbiology, Soochow University, Taipei, Taiwan; 2 Department of Traditional Chinese Medicine, Chang Gung Memorial Hospital, Keelung, Taiwan; 3 Department of Nutrition and Health Sciences, Chang Gung University of Science and Technology, Kweishan, Taoyuan Taiwan; 4 Research Center for Food and Cosmetic Safety and Center for Drug Research and Development, Chang Gung University of Science and Technology, Kweishan, Taoyuan Taiwan; 5 Aesthetic Medical Center, Department of Dermatology, Chang Gung Memorial Hospital, Linkou, Taiwan; 6 Department of Pharmaceutics, College of Pharmacy, Prince Sattam Bin Abdulaziz University, Al Kharj, Saudi Arabia; 7 Pharmaceutics Laboratory, Graduate Institute of Natural Products, Chang Gung University, 259 Wen-Hwa 1st Road, Kweishan, Taoyuan 333 Taiwan; 8 Department of Anesthesiology, Chang Gung Memorial Hospital, Kweishan, Taoyuan Taiwan

**Keywords:** Glycoside, Quercitrin, Isoquercitrin, Rutin, Atopic dermatitis, Skin absorption

## Abstract

Atopic dermatitis (AD) is a multifaceted inflammatory skin condition characterized by the involvement of various cell types, such as keratinocytes, macrophages, neutrophils, and mast cells. Research indicates that flavonoids possess anti-inflammatory properties that may be beneficial in the management of AD. However, the investigation of the glycoside forms for anti-AD therapy is limited. We aimed to assess the ability of quercetin-3-*O*-glycosides in treating AD-like lesions through in silico-, cell-, and animal-based platforms. The glycosylated flavonols of quercitrin, isoquercitrin, and rutin were used in this study. We also tried to understand the influence of glycone type on the bioactivity and skin delivery of glycosides. The glycosides effectively reduced the overexpression of proinflammatory effectors such as interleukin (IL)-6, chemokine (C-X-C motif) ligand (CXCL)1, CXCL8, regulated upon activation normal T cell expressed and secreted (RANTES), and thymus and activation-regulated chemokine (TARC) in the activated keratinocytes. This reduction could be due to the inhibition of extracellular signal-regulated kinase (ERK) and p38 phosphorylation. Isoquercitrin (but not quercitrin and rutin) could arrest the upregulated IL-6 and CCL5 in the macrophage model. The glycosides significantly prevented histamine release from RBL-2H3 cells. The skin absorption examination showed a greater permeation of quercitrin and isoquercitrin than rutin with dual sugar moieties due to the smaller molecular volume and higher lipophilicity. The skin deposition of quercitrin and isoquercitrin was enhanced by about 11-fold in the stripped and delipidized skins, which mimicked AD lesions. The in vivo dinitrochlorobenzene (DNCB)-induced AD mouse model demonstrated less erosion, scaling, and epidermal hyperplasia after topical isoquercitrin treatment. The concentration of cytokines/chemokines in the lesion was decreased by isoquercitrin. These effects were similar to those of tacrolimus ointment. The immunohistochemistry (IHC) displayed the reduction of epidermal hyperproliferation and immune cell infiltration by topical isoquercitrin. The results indicated that the delivery of quercetin glycosides could provide an efficient and safe way to treat AD inflammation.

## Introduction

Atopic dermatitis (AD) is a chronic autoimmune skin disease involving genetic, immunologic, and environmental factors. It is ranked first among cutaneous disorders when estimated in disability-adjusted life-years [1]. The prevalence of AD is 15%‒30% and 2%‒10% for children and adults, respectively [2]. The immune responses of inflammatory mediators such as T helper (Th)1, Th2, and Th17 have been identified in AD pathogenesis [3]. The clinical features of AD lesions are erythema, xerosis, oozing, crusting, increased epidermal thickness, and immune cell infiltration. AD can precede the development of the atopic march, including asthma, allergic rhinitis, and food allergies [4]. Topical treatment is feasible for mild-to-moderate AD patients or patients who cannot tolerate systemic treatment. The representative topical treatment is steroids. However, their application is limited to short-term usage due to side effects and resistance development. The discovery of new anti-AD agents for topical delivery is therefore urgent. Recently, some natural products have attracted increasing attention for the development of AD therapeutics. It has been proven that natural compounds such as flavonoids, alkaloids, and terpenes are effective and safe for AD treatment due to their anti-inflammatory activity to suppress cytokines and chemokines [5].

**Fig. 1:**
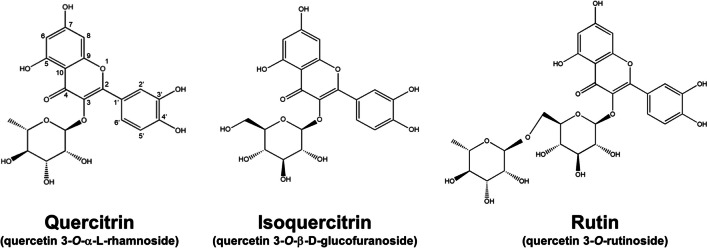
The chemical structures of quercitrin, isoquercitrin, and rutin

One natural anti-AD compound is quercetin, which belongs to flavonol family and has antioxidative and anti-inflammatory activities [6]. Quercetin is found in most vegetables and fruits, such as onions, apples, nuts, and cherry wine. This flavonol can inhibit the activation of keratinocytes, basophils, and mast cells via the arrest of interleukin (IL)−4 and IL-13 in AD-like cell models [7]. Quercetin and quercetin-rich dodder seed extract are also effective for attenuating lesional severity in AD-like mouse models and AD patients, respectively [8, 9]. There are five hydroxyl moieties in the quercetin structure. Glycosylation can occur on all hydroxyl groups to produce glycoside forms by binding sugars [10]. Rhamnoside, glucoside, and rutinoside are the most common glycones of the flavonoid glycoside family and can be found in a broad range of plants. Flavonoid glycosides are known to possess bioactivity against skin inflammation and photoaging [11]. For quercetin, the 3-OH site is the most common target for glycosylation [12]. Quercitrin (quercetin-3-*O*-rhamnoside), isoquercitrin (quercetin-3-*O*-glucoside), and rutin (quercetin-3-*O*-rutinoside) are the major glycosidic forms of quercetin (Fig. 1). These glycosides have been reported to exhibit a suppressive effect on allergic asthma [13]. Anti-AD activity can be expected for these three glycosylated flavonols. Our previous study [14] demonstrated the significantly lower skin absorption of quercetin than its glycosides. Thus, quercetin glycosides may be beneficial as a topically applied agent for treating AD. Quercitrin, isoquercitrin, and rutin are the main bioactive compounds in *Houttuynia cordata* Thunb [15]. *H. cordata* extract manifests antioxidative, anti-inflammatory, and photoprotective activities for treating skin diseases, including AD and photoaging [16–18].

Quercetin has been the subject of many biological investigations. Most studies of the structure–activity relationship (SAR) for quercetin derivatives have focused on the A, B, or C rings. In contrast, fewer reports about the bioactivity of quercetin glycosides exist. Glycosylation of quercetin with different sugars can elicit functions that differ from the parent compound. The aim of this study was to explore and compare the anti-AD activity of quercitrin, isoquercitrin, and rutin. We intended to establish the SAR and structure-permeation relationship (SPR) of the glycosylated flavonols for selecting the potential candidates for anti-AD therapies.

Several cells are interconnected during the progression of AD. The inflammatory response of AD lesions is associated with the stimulation of T cells, keratinocytes, macrophages, mast cells, and basophils [19]. Simultaneous inhibition of different targets in multiple cells is favorable to mitigate AD. In this study, keratinocyte, macrophage, and mast cell models were employed to evaluate the anti-AD activity of the glycosides. The representative cell lines used for the anti-inflammatory study were HaCaT (human keratinocytes), differentiated THP-1 (human monocytes), and RBL-2H3 (rat basophilic leukemia). The in vivo anti-AD effect was examined by using a dinitrochlorobenzene (DNCB)-induced AD-like model in mice. DNCB can induce Th2-mediated immune response that is similar with the immune dysregulation seen in human AD [20]. DNCB activation also simulates the barrier disintegration and chronic inflammation found in human AD [21]. The cytokine profile shown in DNCB-treated mice such as IL-4 and IL-13 closely resembles the cytokine environment observed in human AD [22]. These features make this model an ideal option for investigation into anti-AD therapy.

## Materials and Methods

### In silico* Molecular Modeling*

The physicochemical properties of glycosylated quercetin were assessed utilizing the molecular modeling software Discovery Studio 2021 (Dassault Systems). This software facilitated the calculation of various physicochemical characteristics, such as molecular volume, partition coefficient (log P), total polar surface area, and the number of hydrogen bonds (H-bonds). Additionally, Discovery Studio was employed to analyze the interactions between the glycosides and the lipids present in the stratum corneum (SC). A docking simulation was performed to evaluate the ligand-binding interactions, as indicated by the negative CDOCKER score, between the glycosides and the lipids.

### Capacity Factor Measured by High-Performance Liquid Chromatography

The high-performance liquid chromatography (HPLC) setup for analyzing the quercetin glycosides (ChemFacts) was a Hitachi series-2 HPLC system equipped with a Merck LiChrospher reverse phase C18 column. The mobile phase comprised acetonitrile and water with 0.5% acetic acid (25:75), with a flow rate of 1 ml/min and an ultraviolet detector at 260 nm. The capacity factor (log K’), an index of lipophilicity, was measured from the HPLC. The log *K’* was calculated using the formula log[t_r_-t_0_/t_0_], where t_0_ and t_r_ represented the solvent peak and the glycoside signal retention times, respectively.

### Cell Culture and Treatment

The HaCaT and RBL-2H3 cell lines were cultured in Dulbecco’s Modified Eagle Medium (DMEM) enriched with 10% fetal bovine serum (FBS) and 100 U/ml of penicillin–streptomycin, maintained at 37 °C in a humidified incubator with 5% carbon dioxide. The THP-1 cells were sustained in RPMI 1640 medium supplemented with FBS and penicillin–streptomycin. To differentiate the THP-1 cells into macrophage-like cells, they were treated with phorbol myristate acetate (100 ng/ml) for a duration of 36 h, followed by a 10-h incubation in fresh medium [23]. HaCaT cells were plated in a 96-well plate at a density of 1 × 10^5^ cells/ml and cultured for 24 h. Subsequently, the cells were activated with a combination of tumor necrosis factor (TNF)-α (20 ng/ml) and interferon (IFN)-γ (20 ng/ml) prior to glycoside treatment [24]. After 24 h of culture, an enzyme-linked immunosorbent assay (ELISA) was performed to assess the levels of cytokines and chemokines. For the differentiated THP-1 cells, imiquimod (IMQ, 10 μg/ml) was employed to stimulate the cells at a concentration of 5 × 10^5^ cells/ml [25]. The subsequent procedures for detecting proinflammatory mediators were consistent with those utilized for the HaCaT cells.

### Cytotoxicity of the Quercetin Glycosides

The assessment of cell viability for HaCaT, differentiated THP-1, and RBL-2H3 cell lines following treatment with glycosylated quercetin compounds was conducted utilizing a 3-(4,5-dimethylthiazol-2-yl)−2,5-diphenyltetrazolium bromide (MTT) assay. The cells were subjected to various concentrations (ranging from 0 to 1000 μM) of the glycosides for a duration of 24 h within a carbon dioxide incubator. Subsequent to the incubation period, an MTT solution (0.5 mg/ml) was added to the wells and incubated for an additional hour. The medium was subsequently removed, and the resultant formazan crystals were solubilized in dimethyl sulfoxide. Cell viability was quantified by measuring the absorbance at 570 nm using a microplate reader.

#### ELISA

The protein level of the cytokines and chemokines released by the keratinocytes and macrophages were evaluated by a commercial ELISA kit. The kits were used to analyze the concentrations of IL-6 (BioLegend, Cat. No. 430504), TNF-α (BioLegend, Cat. No. 430204), chemokine (C-X-C motif) ligand (CXCL)1 (R&D Systems, Cat. No. DY275-05), CXCL8 (BioLegend, Cat. No. 431504), regulated upon activation normal T cell expressed and secreted (RANTES, BioLegend, Cat. No. 440804), and thymus and activation-regulated chemokine (TARC, BioLegend, Cat. No. 441104) according to the manufacturer’s instructions.

### Immunoblot Assay

The protein expression levels of mitogen-activated protein kinases (MAPKs) including c-Jun N-terminal kinase (JNK, Cell signaling, Cat. No. 9252S), extracellular signal-regulated kinase (ERK, Cell signaling, Cat. No. 9102S), and p38 (Cell signaling, Cat. No. 9212S) as well as signal transducer and activator of transcription (STAT)3 (Cell signaling, Cat. No. 4904S) in human keratinocytes were assessed utilizing Western blotting techniques. The keratinocytes were subjected to lysis with a lysis buffer, and the total protein concentration was determined via a Bradford assay at a wavelength of 595 nm. Subsequently, the proteins were separated by sodium dodecyl sulfate–polyacrylamide gel electrophoresis (SDS-PAGE) and transferred to a polyvinylidene fluoride (PVDF) membrane. This membrane was incubated overnight at 4 °C with a primary antibody diluted to 1:1000. After the incubation period, the membrane was rinsed with tris-buffered saline. The protein signals were visualized using a horseradish peroxidase-conjugated secondary antibody diluted to 1:5000, in conjunction with a Western Lightning Plus-ECL detection kit. Signal detection was performed using a ChemiDoc imaging system (Bio-Rad). Densitometric analysis was subsequently conducted to quantify the protein concentrations.

### Chemotaxis Assay

The chemotaxis assay evaluated the keratinocyte stimulation induced by the conditioned medium from differentiated THP-1 [24]. The conditioned medium was collected from THP-1 cells activated by IMQ (10 μg/ml) and treated with glycosides at concentrations of 0‒10 μM for 2 h. Then, the medium was added to the keratinocytes (6 × 10^5^ cells/ml) in a 96-well plate. The plate was incubated at 37 °C for 15 min before the immunoblotting assay. The protein amount of STAT3 in the HaCaT cells was detected to appraise the chemotaxis between the keratinocytes and macrophages.

### Degranulation of RBL-2H3 Cells Determined by Histamine and β-Hexosaminidase Release

The histamine level of the RBL-2H3 cells was evaluated to determine the degradation. The RBL-2H3 cells (1 × 10^5^ cells/ml) were seeded in a 24-well plate and primed with anti-2,4-dinitrophanylated (DNP) immunoglobulin (Ig)E (50 ng/ml in 30% FBS) for 24 h [26]. The sensitized RBL-2H3 was treated with quercetin glycosides at 0‒10 μM for 1 h, followed by activation with DNP-bovine serum albumin (BSA) at 100 ng/ml for 4 h. The histamine concentration was analyzed using an ELISA kit based on the manufacturer’s instructions. For the β-hexosaminidase assay, the cells were collected and centrifuged at 15 x*g* at 4 °C for 5 min after the stimulation and glycoside intervention. The supernatant was incubated for 1 h with 4-nitrophenyl-N-acetyl-β-glucosaminide (1 mM in 0.1 M citrate buffer). We stopped this reaction through the incorporation of 0.1 M Na_2_CO_3_-NaHCO_3_. The absorbance at 405 nm was detected by using a microplate reader.

### Animals

The one-week-old pigs were acquired from PigModel Animal Technology located in Miaoli, Taiwan. Male Balb/c mice, aged between six to eight weeks, were sourced from the National Laboratory Animal Center in Taipei, Taiwan. All animal experimentation was carried out in strict adherence to the Guidelines for the Care and Use of Laboratory Animals established by Chang Gung University. The experimental protocol received approval from the Institutional Animal Care and Use Committee (CGU111-174).

### In vitro* Permeation Test*

The transdermal absorption of glycosylated flavonols was assessed utilizing Franz diffusion cells with excised porcine skin. The dorsal skin was positioned between the donor and receptor compartments of the Franz cells, ensuring that the stratum corneum (SC) faced the donor compartment. The effective penetration area of the cells was measured at 0.79 cm^2^. In addition to utilizing intact skin, SC-stripped and delipidized skin samples were prepared for the in vitro permeation test (IVPT) in accordance with the methodology established in prior research [27]. The donor compartment was filled with 0.5 ml of glycosides (5 mM) dissolved in a 20% propylene glycol (PG) solution buffered to pH 7.4, while the receptor compartment (5 ml) contained a 30% ethanol solution buffered to pH 7.4. The temperature of the receptor compartment was maintained at 37 °C, with a stirring rate of 600 rpm. Aliquots of 0.3 ml from the receptor medium were collected at specified time intervals. At the conclusion of the 24-h experiment, the skin was extracted from the cells to quantify the glycoside content within the skin reservoir. The skin samples were subsequently weighed, sectioned, and homogenized using a MagNA Lyser in methanol. The resultant homogenate was then centrifuged and filtered to isolate the supernatant. HPLC was employed to quantify the glycoside concentrations in both skin deposition (nmol/mg) and receptor accumulation (nmol/cm^2^). The flux (nmol/cm^2^/h) was calculated based on the slope of the cumulative amount-time curve representing receptor accumulation. Furthermore, the dermal/transdermal selectivity index (S value) was determined using the formula for skin deposition divided by flux.

### AD-Like Animal Model

Twenty-four mice were divided into four groups: healthy control, DNCB stimulation with topical administration of PBS, DNCB stimulation with topical administration of isoquercitrin, and DNCB stimulation with topical administration of tacrolimus ointment (Protopic) as the positive control. The dorsal area of the mouse skin was sensitized with 1% DNCB (0.1 ml) in a mixture of acetone and olive oil (4:1) for three consecutive days (day 0, 1, and 2). After five days of sensitization, the skin was challenged with 0.1 ml of 0.5 DNCB on day 7, 9, 11, and 13. Isoquercitrin (20 mM in 20% PG/pH 7.4 buffer, 0.15 ml) or tacrolimus ointment (0.15 g) was topically applied to the dorsal skin from day 7 to day 13. Transepidermal water loss (TEWL) was measured daily using a Cutometer MPA580 (Courage and Khazaka). The skin surface was monitored using a digital camera and microscopy (AM7515M2T, Dino-Lite) on day 14. After sacrificing the mice on day 14, the treated skin was removed to analyze the proinflammatory mediators. We excised the treated skin with an area of about 1 × 1 cm^2^. The skin with a weight of 4 mg was extracted with PBS and homogenized by a MagNA Lyser for the ELISA test. The total protein concentration of the samples was determined by Bradford reagent (Sigma-Aldrich, Cat No. B6916) for calibration of the cytokine/chemokine amount in the skin.

### Histological Observation

The skin excised from the in vivo study was fixed in a 10% formalin solution and then embedded in paraffin. The skin was cut into a 5-μm thickness for hematoxylin and eosin (H&E) staining. In addition, the skin slices were subjected to immunohistochemistry (IHC) by incubating them with the antibody against Ki67, F4/80, or Ly6G (diluted at a ratio of 1:100). The slices were then incubated with biotinylated donkey anti-goat IgG for 20 min. We used a Leica DMi8 microscope to visualize the skin sections.

### Skin Irritation Assay

The skin irritation test was performed to examine whether isoquercitrin could induce toxicity on healthy mouse skin. The formulation for isoquercitrin was the same as that used in the AD-like mice. Topical isoquercitrin was applied on the dorsal skin for five consecutive days, with the formulation being renewed daily. The skin appearance, pH, and TEWL were examined daily. The skin was excised for H&E staining on day 5.

### Statistical Assay

The data in the present work were presented as the mean and standard error of the mean (SEM). The Kruskal–Wallis test was employed to analyze the significance of variations among the various treatment groups. Dunn’s test was used as a post hoc test to identify the differences. Statistical significance was determined at probability levels of 0.05, 0.01, and 0.001.

## Results

### Physicochemical Features of Quercetin Glycosides

The physicochemical properties of quercitrin, isoquercitrin, and rutin were computed using in silico molecular modeling. Rutin with dual sugar moieties showed a larger molecular size, as evidenced by the molecular weight (MW) and molecular volume, compared to the glycosides with a single sugar (Table 1). The log *P* is an indicator of lipophilicity. Because of the presence of glycones in the structure, all compounds exhibited significantly low log *P* (< 0.6), demonstrating a hydrophilic specialty. This log *P* was lower than the parent aglycone compound quercetin (1.8 as calculated by Discovery Studio 2021). The log *K’* is another lipophilicity indicator that can be calculated from the HPLC chromatogram through a reverse phase column. Similar to the log *P*, the log *K’* decreased with a tendency of quercitrin > isoquercitrin > rutin. The total polarity surface is the molecular hydrophilicity estimated by the surface sum of all polar atoms. The tendency of total polarity surface of the three compounds was opposite to that of the log *P* and log *K’*. Due to the abundance of hydroxyl groups in the structure, the glycosides possessed many H-bond acceptor and donor numbers, with rutin showing the largest amount.

**Table 1:** Physicochemical properties of the flavonol glycosides from *Houttuynia caodata* estimated by molecular modeling

Compound	MW^a^ (Da)	Molecular volume (Å^3^)^b^	log *P*^c^	log *K’*^d^	Total polarity surface	H-bond acceptor number	H-bond donor number
Quercitrin	448.38	328.59	0.59	0.11	186.37	11	7
Isoquercitrin	464.38	329.27	−0.30	−0.28	206.60	12	8
Rutin	610.52	440.41	−1.16	−0.53	265.52	16	10

^a^ MW, molecular weight

^b^ Molecular volume was calculated by molecular modeling

^c^ log *P*, n-octanol/water partition coefficient

^d^ log *K’*, logarithm of (tr − t0)/t0, tr is the retention time of compound peak, t0 is the retention time of solvent peak

### The Effect of Glycosides on Cytokine/Chemokine Inhibition in Keratinocytes

**Fig. 2:**
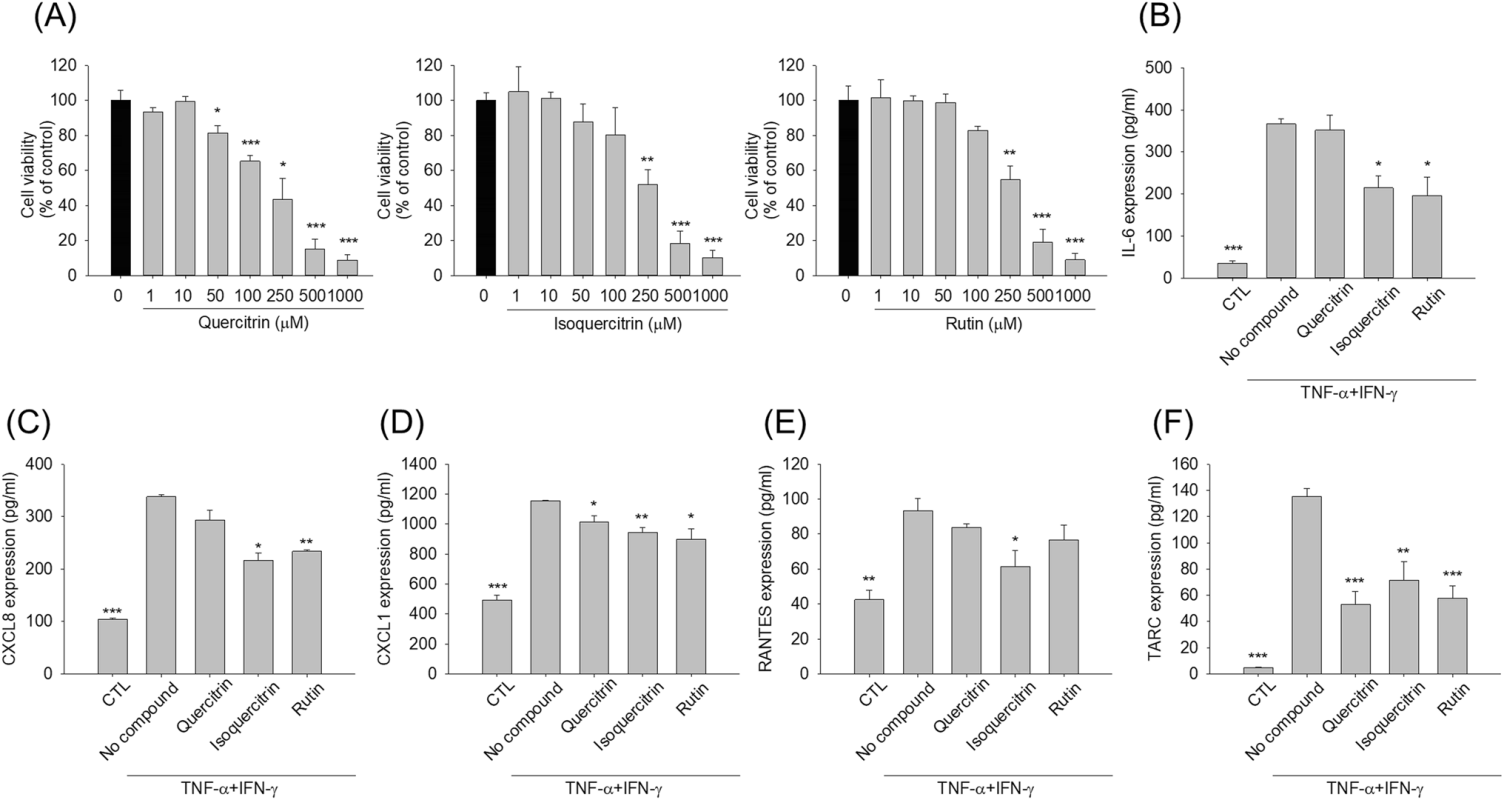
The effect of quercetin glycosides on cytokine/chemokine in TNF-α/IFN-γ-activated keratinocytes. (A) The cell viability of quercetin glycosides at 0 − 1000 μM after a 24-h treatment. (B) The IL-6 expression in TNF-α/IFN-γ-activated HaCaT after the treatment of quercetin glycosides at 10 μM after a 24-h treatment. (C) The CXCL8 expression in TNF-α/IFN-γ-activated HaCaT after the treatment of quercetin glycosides at 10 μM after a 24-h treatment. (D) The CXCL1 expression in TNF-α/IFN-γ-activated HaCaT after the treatment of quercetin glycosides at 10 μM after a 24-h treatment. (E) The RANTES expression in TNF-α/IFN-γ-activated HaCaT after the treatment of quercetin glycosides at 10 μM after a 24-h treatment. (F) The TARC expression in TNF-α/IFN-γ-activated HaCaT after the treatment of quercetin glycosides at 10 μM after a 24-h treatment. All experiments were performed three times (*n* = 3). Data are represented as mean ± SEM. *P < 0.05, **P < 0.01, and ***P < 0.001 when compared to the group of non-treatment control or TNF-α/IFN-γ treatment alone

We performed an MTT assay to check the cytotoxicity of the glycosylated quercetins in the keratinocytes. Quercitrin did not show cytotoxicity under 10 μM (Fig. 2A). The cell viability was concentration-dependent in the case of quercitrin at doses greater than 50 μM. Isoquercitrin and rutin had no cytotoxic effect at concentrations ≤ 100 μM, but had a partial effect at concentrations > 100 μM. Hence, the keratinocytes were treated with 10 μM of compounds in the following experiments. Subsequently, we investigated the effect of the glycosides on TNF-α/IFN-γ-induced keratinocyte activation. TNF-α/IFN-γ significantly upregulated the protein level of IL-6, CXCL8, CXCL1, RANTES, and TARC (Fig. 2B to 2F). Isoquercitrin and rutin, but not quercitrin, reduced the secretion of IL-6 and CXCL8 in the activated cells (Fig. 2B and 2C). CXCL1 concentration in the activated keratinocytes was about two-fold higher than the normal cells (Fig. 2D). The CXCL1 level decreased by 12%, 18%, and 22% in the quercitrin, isoquercitrin, and rutin groups compared to the activation group, respectively. A comparable reduction was found between isoquercitrin and rutin. Isoquercitrin treatment significantly decreased RANTES concentration by 34% versus the stimulated group (Fig. 2E). Quercitrin and rutin revealed no effect on RANTES overexpression. All glycosides significantly downregulated TARC secretion by two- to three-fold, as compared to the TNF-α/IFN-γ group (Fig. 2F).

### The Effect of Glycosides on the Phosphorylation of MAPKs

**Fig. 3:**
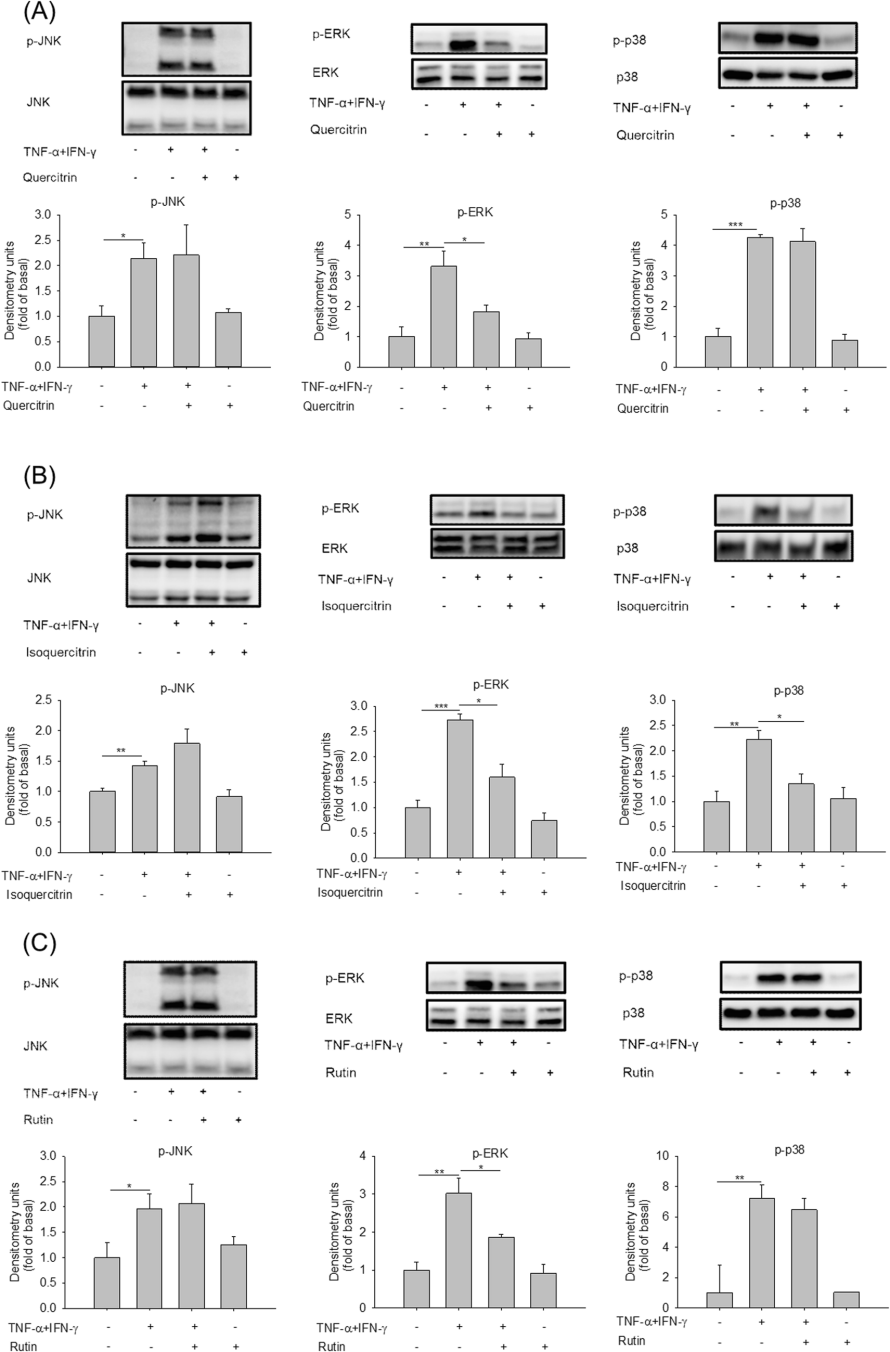
The effect of quercetin glycosides on MAPK signaling pathways in keratinocytes. (A) The p-MAPK/total MAPK expression in TNF-α/IFN-γ-activated HaCaT after the treatment of quercitrin at 10 μM after a 24-h treatment. (B) The p-MAPK/total MAPK expression in TNF-α/IFN-γ-activated HaCaT after the treatment of isoquercitrin at 10 μM after a 24-h treatment. (C) The p-MAPK/total MAPK expression in TNF-α/IFN-γ-activated HaCaT after the treatment of rutin at 10 μM after a 24-h treatment. All experiments were performed three times (*n* = 3). Data are represented as mean ± SEM. *P < 0.05, **P < 0.01, and ***P < 0.001 when compared to the group of TNF-α/IFN-γ treatment alone

TNF-α/IFN-γ-activated MAPK signaling pathways are known to be involved in AD. The expression level of MAPKs, including JNK, ERK, and p38 in keratinocytes were investigated using immunoblot analysis. We showed that TNF-α/IFN-γ stimulated the phosphorylation of MAPKs (Fig. 3). For quercitrin treatment at 10 μM, no significant effect on p-JNK and p-p38 was observed in the stimulated keratinocytes (Fig. 3A). On the other hand, treatment of keratinocytes with quercitrin significantly attenuated the TNF-α/IFN-γ-induced phosphorylation of ERK. The same as quercitrin, isoquercitrin showed negligible effects on the p-JNK level in the cells (Fig. 3B). The treatment of isoquercitrin at 10 μM abrogated p-ERK and p-p38 at a significant level. In the case of rutin, ERK phosphorylation, but not JNK and p38, was reduced in the activated keratinocytes (Fig. 3C). The result of the MAPKs suggested the anti-inflammatory activity of quercetin glycosides, potentially mediated through the suppression of ERK signaling.

### The Effect of Glycosides on the Activation of Macrophages

**Fig. 4:**
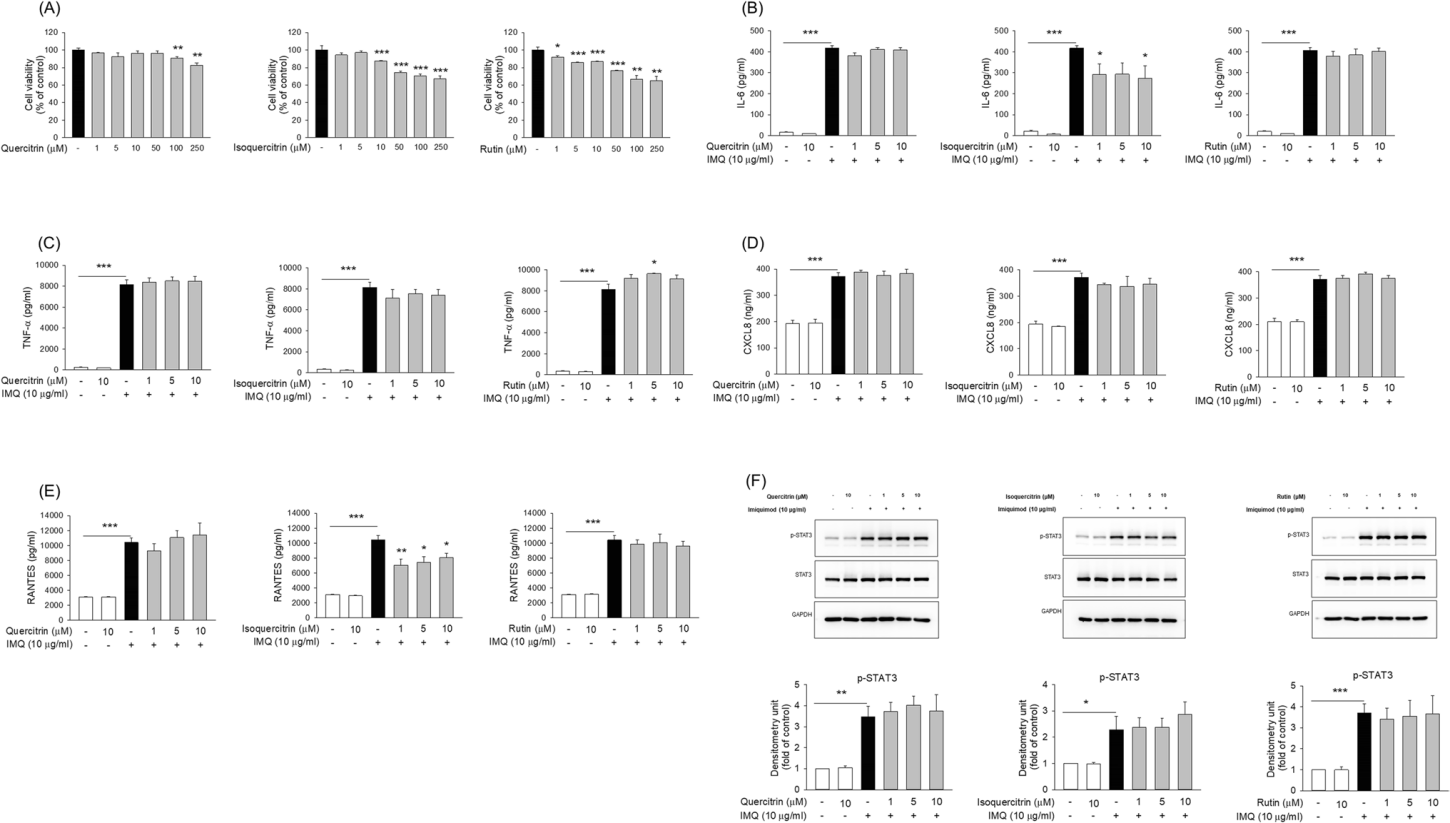
The effect of quercetin glycosides on cytokine/chemokine in IMQ-activated THP-1 cells. (A) The cell viability of quercetin glycosides at 0 − 250 μM after a 24-h treatment. (B) The IL-6 expression in IMQ-activated THP-1 after the treatment of quercetin glycosides at 0‒10 μM after a 24-h treatment. (C) The TNF-α expression in IMQ-activated THP-1 after the treatment of quercetin glycosides at 0‒10 μM after a 24-h treatment. (D) The CXCL8 expression in IMQ-activated THP-1 after the treatment of quercetin glycosides at 0‒10 μM after a 24-h treatment. (E) The RANTES expression in IMQ-activated THP-1 after the treatment of quercetin glycosides at 0‒10 μM after a 24-h treatment. (F) Western blotting of the phosphorylation of STAT3 in HaCaT cells treated with quercetin glycosides at 0‒10 μM, incubated with the conditioned medium of THP-1 cells for 24 h, was performed for p-STAT3 expression. All experiments were performed three times (*n* = 3). Data are represented as mean ± SEM. *P < 0.05, **P < 0.01, and ***P < 0.001 when compared to the group of non-treatment control or IMQ treatment alone

Macrophages are one of the antigen-presenting cells involved in AD inflammation. We established a differentiated THP-1 cell model as the macrophage platform to explore the effect of glycoside on AD-induced inflammation. At first, the differentiated THP-1 was exposed to various doses (0‒250 μM) of glycoside compounds to test the cytotoxicity. Quercitrin and isoquercitrin exhibited no cytotoxicity at amounts up to 50 μM in the macrophages (Fig. 4A). Isoquercitrin showed a dose-dependent decrease in macrophage viability, with maintenance of viability exceeding 80% at doses ≤ 10 μM. Similar to isoquercitrin, the viability could be maintained at 80% for rutin exposure at ≤ 10 μM. Further experiments of pro-inflammatory mediator detection were conducted at glycoside concentrations of 1, 5, and 10 μM at > 80% viability. The representative mediators studied here were IL-6, TNF-α, CXCL-8 and RANTES. IMQ intervention of the macrophages displayed increased amount of these cytokines/chemokines compared to that in the control group (Fig. 4B to 4E). Isoquercitrin, but not quercitrin and rutin, decreased IL-6 upregulation in the THP-1 cells (Fig. 4B). The increased isoquercitrin concentrations (5 and 10 μM) did not further enhance IL-6 inhibition, suggesting that 1 μM was sufficient to induce the inhibitory effect. All glycosides tested showed an insignificant effect on TNF-α and CXCL8 (Fig. 4C and 4D). Isoquercitrin significantly decreased IMQ-induced RANTES production in the THP-1 cells by about 30% compared to the IMQ treatment alone (Fig. 4E). This inhibitory effect was not detected in the treatment of quercitrin and rutin. This result coincided with that of keratinocytes, as only isoquercitrin suppressed the overexpressed RANTES level.

Macrophage chemotaxis mediates the interplay between macrophages and the resident cells. To understand the impact of quercetin glycosides on macrophage-keratinocyte crosstalk, we evaluated the effect of an IMQ-activated macrophage-conditioned medium on keratinocyte proliferation using Western blot analysis. The conditioned medium from the IMQ-stimulated macrophages elicited the elevation of phosphorylation STAT3, a bio marker of keratinocyte proliferation (Fig. 4F). The phosphorylation level of STAT3 in the condition-medium-exposed keratinocytes was unchanged after the glycoside treatment, demonstrating that the glycosides failed to prevent macrophage chemotaxis.

### The Effect of Glycosides on RBL-2H3 Cell Degranulation

**Fig. 5:**
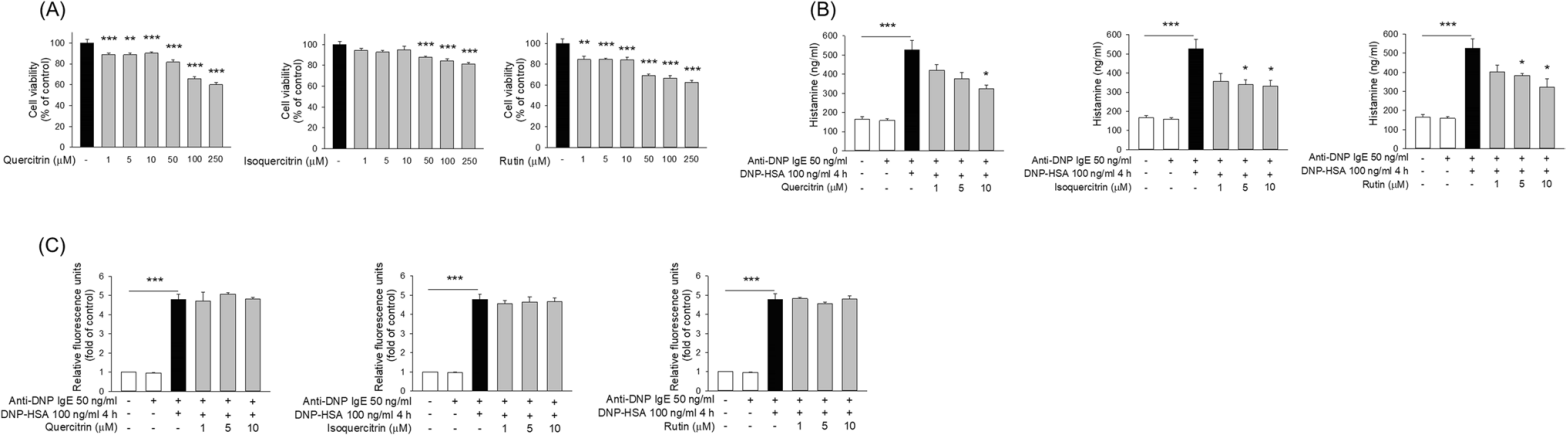
The effect of quercetin glycosides on degranulation in anti-DNP IgE/DNP-HSA-activated RBL-2H3 cells. (A) The cell viability of quercetin glycosides at 0 − 250 μM after a 24-h treatment. (B) The histamine production in anti-DNP IgE/DNP-HSA -activated RBL-2H3 after the treatment of quercetin glycosides at 0‒10 μM after a 24-h treatment. (C) The β-hexosaminidase production in anti-DNP IgE/DNP-HSA -activated RBL-2H3 after the treatment of quercetin glycosides at 0‒10 μM after a 24-h treatment. Data are represented as mean ± SEM. *P < 0.05, **P < 0.01, and ***P < 0.001 when compared to the group of non-treatment control or anti-DNP IgE/DNP-HSA treatment alone

The mast cell degranulation in AD causes pruritus, swelling, and erythema in the lesional skin. In addition, basophils are crucial to AD progression by releasing proinflammatory effectors. The RBL-2H3 cell line can act as a cell model of both mast cells and basophils in AD. To know the inhibitory activity of glycosylated flavonoids on the degranulation of the cells, RBL-2H3 cells were sensitized with IgE and DNP-BSA. All glycosides decreased RBL-2H3 viability in a concentration-dependent manner, with isoquercitrin displaying greater viability than the other compounds (Fig. 5A). All isoquercitrin concentrations generated a viability of > 80%. Quercitrin and rutin at ≤ 50 μM and ≤ 10 μM produced an RBL-2H3 cell viability of > 80%, respectively. The activation of the cells with IgE and DNP-BSA elevated the histamine amount by three-fold as compared to the non-treatment group (Fig. 5B). Treatment with the glycosides effectively suppressed histamine release from cells. The inhibitory effect of the three compounds on histamine release was comparable. This finding highlighted the preventive activity of glycosylated quercetins on mast cell degradation. β-hexosaminidase is a lysosomal enzyme secreted by the granules of mast cells and basophils. IgE/DNP-BSA sensitization significantly enhanced β-hexosaminidase release in RBL-2H3 cells (Fig. 5C). However, all glycosides failed to decrease the β-hexosaminidase induced by IgE plus DNP-BSA.

### The Skin Absorption of Glycosides

**Fig. 6:**
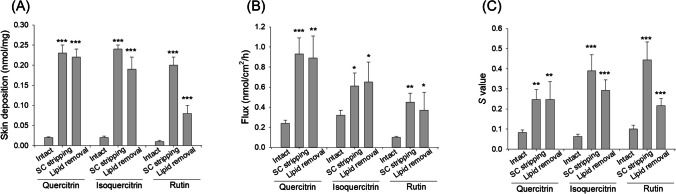
In vitro skin absorption of quercetin glycosides via baby pig skin in an IVPT. (A) Skin deposition at a dose of 5 mM after topical treatment on pig skin for 24 h. (B) The flux at a dose of 5 mM after topical treatment on pig skin for 24 h. (C) The *S* value after topical treatment on pig skin for 24 h. Data are represented as mean ± SEM. *P < 0.05, **P < 0.01, and ***P < 0.001 when compared to the group of intact akin absorption

The cutaneous delivery of the three glycosides was compared through IVPT by using the pig skin as the permeation barrier. The compound deposition in the skin reservoir was first determined. The intact skin depositions of quercitrin and isoquercitrin were two times higher than that of rutin (Fig. 6A). Quercitrin and isoquercitrin revealed the approximate intact skin deposition. No deglycosylation occurred in the skin after glycoside absorption into the skin according to the determination by HPLC. We also removed the SC layer (SC stripping) and the lipids in the SC (lipid removal) to simulate AD-affected skin with a disintegrated barrier function. Both SC stripping and lipid removal increased skin deposition of quercitrin and isoquercitrin by about 11-fold. The removal of SC enhanced rutin deposition by 20-fold, whereas only an eight-fold effect was achieved after lipid removal. We also detected the glycoside delivery across the skin by measuring the compound accumulation in the receptor compartment. Flux was calculated from the slope of the cumulative amount in different collection time points. Since baby pig skin was utilized in this experiment, the flux could be an indicator of the glycoside diffusion into deeper skin strata or systemic circulation. Similar to the data for the intact skin deposition, the flux of quercitrin and isoquercitrin was higher than that of rutin (Fig. 6B). SC stripping and lipid removal exhibited a comparable level to promote the flux of the glycosides. The quercitrin flux of the stripped and delipidized skins was higher than the isoquercitrin flux, although no statistical significance was achieved. Rutin showed minimal flux enhancement after stripping and lipid removal. The *S* value represents the selectivity between skin deposition and flux, with a higher value representing the preferable absorption in the skin and a lower *S* value indicating the preferable delivery to circulation. The *S* value in the intact skin was comparable for the three compounds (Fig. 6C). The *S* value in the stripped skin was increased in the order of rutin > isoquercitrin > quercitrin.

The possible affinity between SC lipids and quercetin glycosides was predicted by in silico molecular docking. The major lipids in SC are ceramides (50%), free fatty acids (15%), cholesterol (25%), and cholesteryl sulfate (5%). The negative CDOCKER derived from the in silico data signifies the interaction strength. Isoquercitrin was the permeant with the strongest interaction with ceramide II (Table 2). A comparable negative CDOCKER of ceramide III was found for the three glycosides. The strongest interaction with ceramide VI was detected for quercitrin, followed by isoquercitrin and rutin. A contrary result was observed in the case of palmitic acid, the main free fatty acid in SC. The interaction with cholesterol and cholesteryl sulfate was either positive or not detected via molecular docking, suggesting a negligible interaction with these lipids.

**Table 2:** The negative CDOCKER energy of the flavonol glycosides from *Houttuynia caodata* to interact with the stratum corneum components determined by molecular modeling

Compound	Ceramide II	Ceramide III	Ceramide VI	Palmitic acid	Cholesterol	Cholesteryl sulfate
Quercitrin	−27.87	−31.88	−31.03	−21.76	-	114.50
Isoquercitrin	−31.48	−31.55	−27.35	−22.50	-	115.49
Rutin	−27.87	−31.75	−24.92	−24.92	48.96	115.49

-, no interaction

### *The Anti-Inflammatory Effect of Isoquercitrin on the *in vivo* AD-like Lesion*

**Fig. 7:**
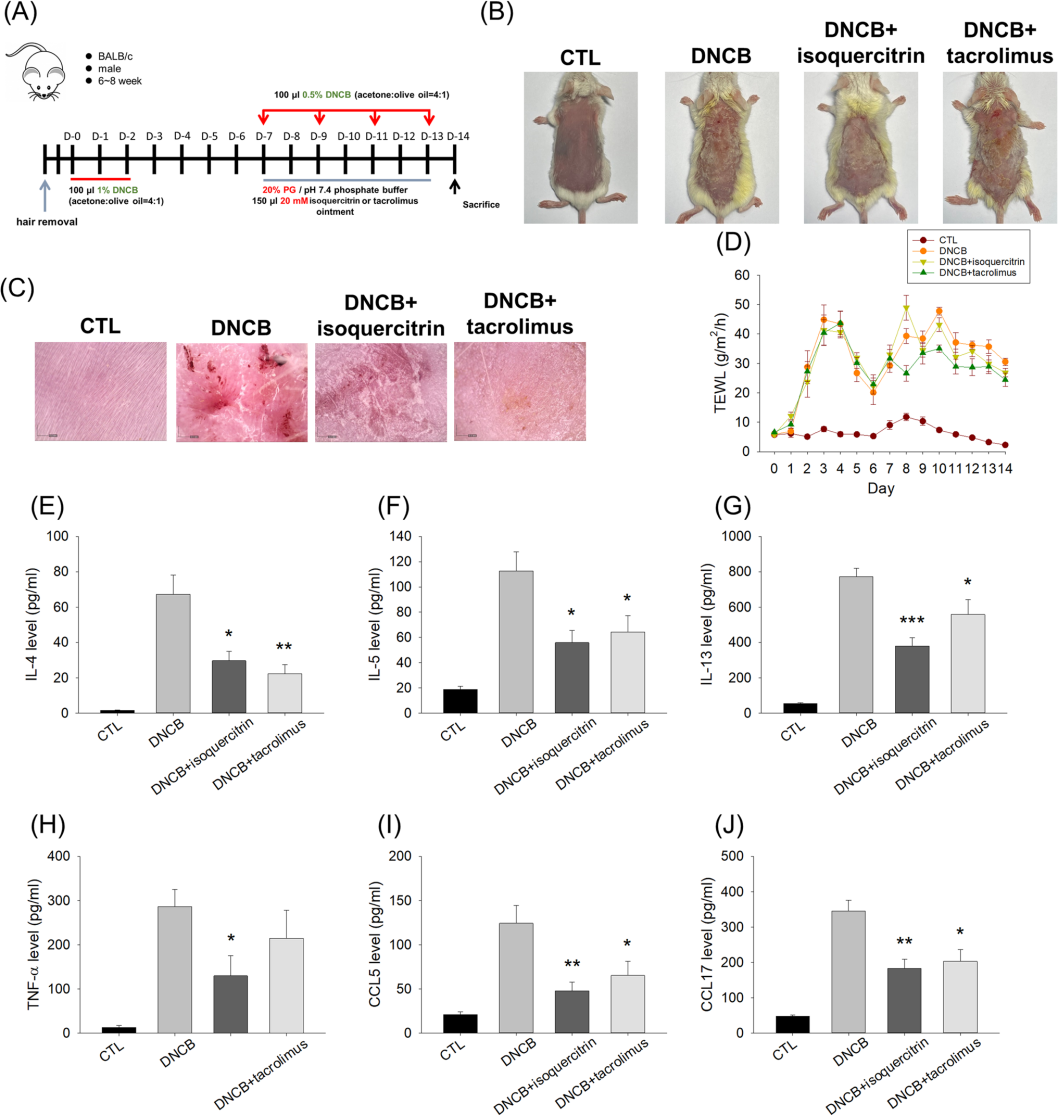
The effect of isoquercitrin and tacrolimus ointment (Protopic) on in vivo DNCB-induced AD-like lesion in the mouse model. (A) The protocol of AD-like lesion induction and compound treatment. (B) Phenotypical images of dorsal skin in AD-like mice after treatment with or without isoquercitrin and tacrolimus on Day 14. (C) Microscopic images of dorsal skin in AD-like mice after treatment with or without isoquercitrin and tacrolimus on Day 14. (D) TEWL measured during 14 days. (E) The expression level of IL-4 in lesional skin on Day 14 determined by ELISA. (F) The expression level of IL-5 in lesional skin on Day 14 determined by ELISA. (G) The expression level of IL-13 in lesional skin on Day 14 determined by ELISA. (H) The expression level of TNF-α in lesional skin on Day 14 determined by ELISA. (I) The expression level of RANTES in lesional skin on Day 14 determined by ELISA. (J) The expression level of TARC in lesional skin on Day 14 determined by ELISA. All experiments were performed in six animals (*n* = 6). Data are represented as mean ± SEM. *P < 0.05, **P < 0.01, and ***P < 0.001 when compared to the group of DNCB treatment alone

The results of in the vitro experiments prompted us to further perform the in vivo study. Among the three compounds tested, isoquercitrin demonstrated the greatest anti-inflammatory activity in the cell-based study. The IVPT showed a facile absorption of isoquercitrin in the barrier-defective skin. This glucose-containing glycoside was chosen to assess the possible anti-AD activity in the DNCB-stimulated mouse model. The experimental scheme for AD induction and isoquercitrin application is illustrated in Fig. 7A. The isoquercitrin dose of 20 mM, the concentration the same as IVPT, was chosen in the in vivo study since this concentration was promised to elicit a facile delivery into the skin based on IVPT results. The two-week sensitization of DNCB caused erythema, edema, scaling, and xerosis, as observed by a digital camera (Fig. 7B). After topical application for seven days, isoquercitrin attenuated the DNCB-induced symptoms in the en face view. This improvement was similar to that visualized in the tacrolimus ointment. The microscopic observation of the skin surface illustrated that atopy was induced by DNCB based on the red, erosive, and scarred appearance (Fig. 7C). The group tested with isoquercitrin showed a noticeable alleviation of the abnormality. The mice administered tacrolimus ointment revealed lower levels of cutaneous erosion compared to the isoquercitrin group. The changes in the skin barrier properties were measured by the TEWL. In the first week before anti-AD treatment, the DNCB challenge significantly increased the TEWL as compared to the healthy control (Fig. 7D). In the second week following the anti-AD treatment, both isoquercitrin and tacrolimus slightly restored the barrier function of the lesional skin.

To understand the proinflammatory mediator production in the lesion, the protein concentration of IL-4, IL-5, IL-13, TNF-α, RANTES, and TARC were analyzed. The amount of these cytokines/chemokines was greatly increased in the skin upon exposure to DNCB (Fig. 7E to 7J). Topical delivery of isoquercitrin abolished DNCB-induced interleukin overexpression by about two-fold (Fig. 7E to 7G). This inhibition level approximated the suppressive effect of the tacrolimus ointment. Treatment with isoquercitrin markedly reduced the DNCB-induced secretion of TNF-α from 287 to 88 pg/ml (Fig. 7H). On the other hand, tacrolimus failed to arrest the overexpressed TNF-α in the lesional site. Both isoquercitrin and tacrolimus significantly decreased the upregulation of the chemokines (RANTES and TARC) induced by DNCB (Fig. 7I and 7J).

**Fig. 8:**
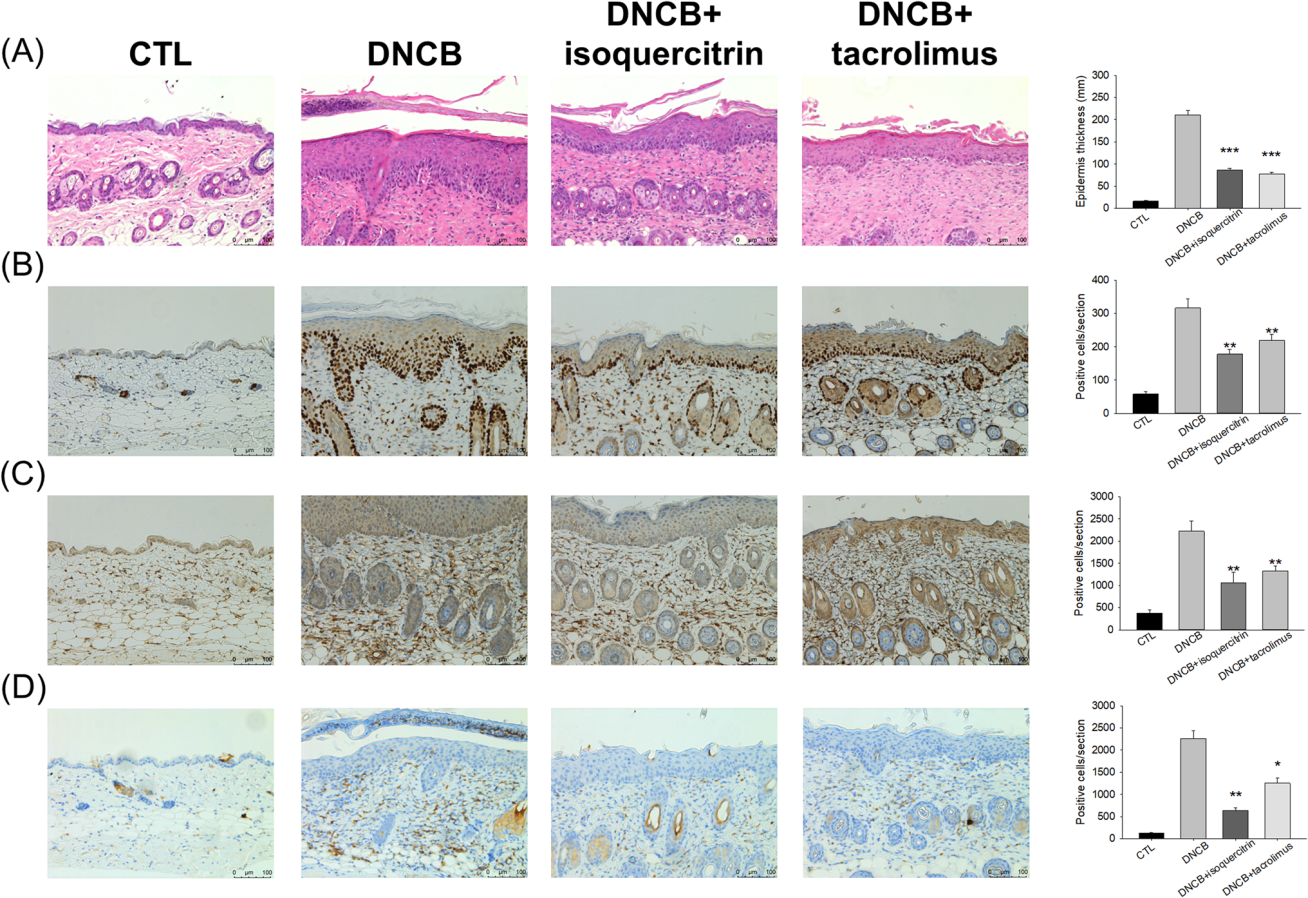
Histological assessment of lesional skin in DNCB-induced AD-like mice after treatment with or without isoquercitrin and tacrolimus ointment (Protopic) on Day 14. (A) H&E-stained skin slices and the epidermal thickness (μm) measurement. (B) Ki67-labeled skin slices and the quantitative analysis of Ki67-positive cells in the skin. (C) F4/80-labeled skin slices and the quantitative analysis of F4/80-positive cells in the skin. (D) Ly6G-labeled skin slices and the quantitative analysis of Ly6G-positive cells in the skin. Data are represented as mean ± SEM. *P < 0.05, **P < 0.01, and ***P < 0.001 when compared to the group of DNCB treatment alone

The control skin without DNCB irritation displayed a normal stratified epidermis with typical keratinization (Fig. 8A). H&E-stained histology illustrated a thick epidermis in the DNCB-treated skin region because of the keratinocyte proliferation. In contrast, the epidermal hyperplasia was decreased by isoquercitrin and tacrolimus compared to the DNCB treatment alone. The epidermal thickness was 16 μm in the healthy group, and this increased to 211 μm in the group exposed to DNCB alone (right panel of Fig. 8A). This increase was reduced to 87 and 77 μm by isoquercitrin and tacrolimus, respectively. The IHC of the skin section with the Ki67 antibody verified that DNCB evoked an aberrant proliferation of keratinocytes in the basal layer of the epidermis (Fig. 8B). Isoquercitrin and tacrolimus notably reduced this abnormal change. DNCB-induced Ki67 overexpression was inhibited by 44% and 31% after topical delivery of isoquercitrin and tacrolimus, respectively (right panel of Fig. 8B). IHC analysis of the F4/80 and Ly6G antibodies was used to investigate the effect of isoquercitrin on the infiltration of macrophages and neutrophils, respectively. The results of the F4/80 IHC showed that the number of macrophages in the dermis was significantly increased after repeated stimulation with DNCB (Fig. 8C). The isoquercitrin-treated group decreased the infiltration of macrophages. The F4/80-stained region in the isoquercitrin-treated skin was about two times lower than that of the DNCB treatment alone and showed no statistically significant difference compared to the tacrolimus group (right panel of Fig. 8C). Ly6G-positive staining increased considerably in the dermis of the mice challenged with DNCB, which decreased substantially upon the skin delivery of isoquercitrin (Fig. 8D). The recruitment of Ly-6G-positive cells by DNCB was partially reduced upon the application of tacrolimus. The Ly-6G-positive staining in the groups treated with isoquercitrin and tacrolimus was 72% and 45% lower than that in the DNCB treatment group (right panel of Fig. 8D).

### Skin Irritation Assay

**Fig. 9:**
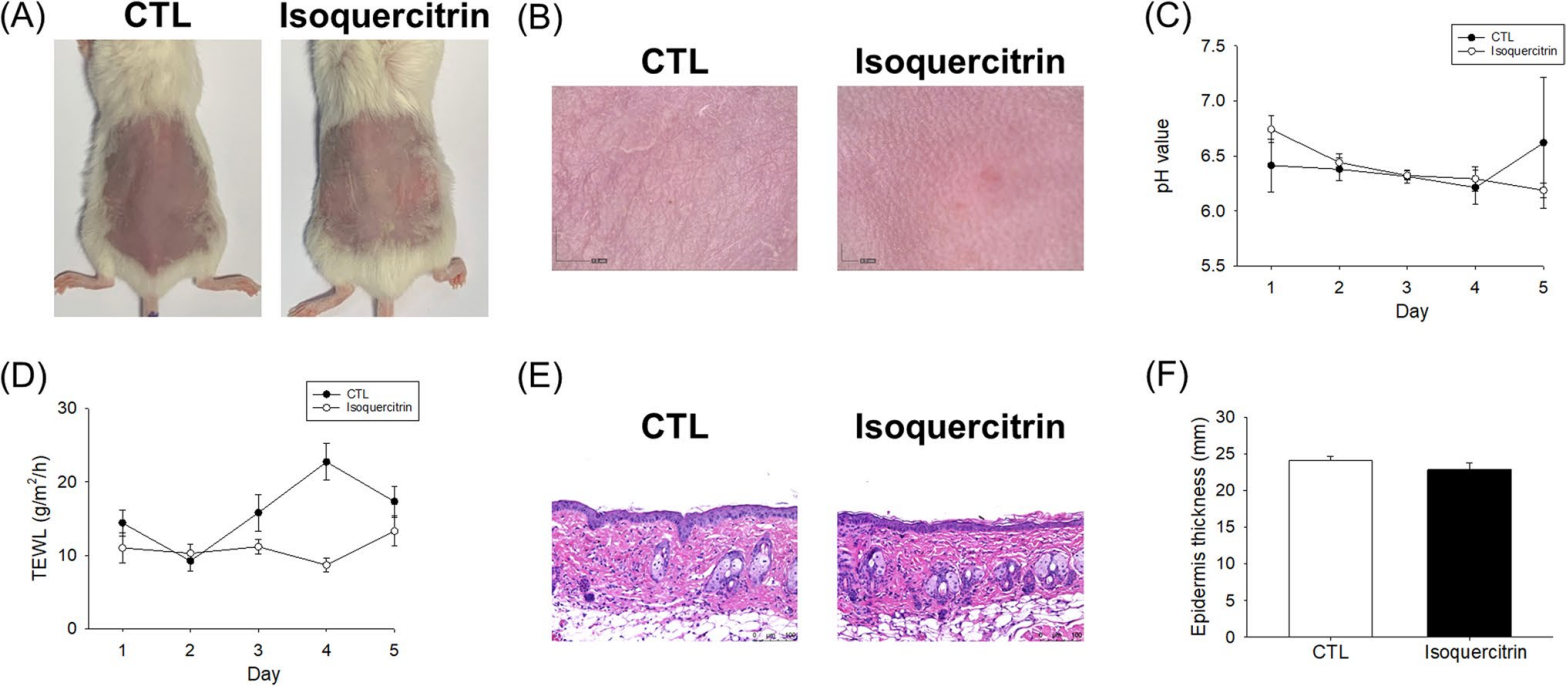
Skin tolerance examination of healthy mouse skin by five consecutive day treatment of topically applied isoquercitrin. (A) Phenotypical images of dorsal skin in healthy mice after topical treatment with or without isoquercitrin on Day 5. (B) Microscopic images of dorsal skin in healthy mice after topical treatment with or without isoquercitrin on Day 5. (C) The pH change of dorsal skin in healthy mice after topical treatment with or without isoquercitrin during 5 days. (D) The TEWL change of dorsal skin in healthy mice after topical treatment with or without isoquercitrin during 5 days. (E) H&E-stained slices of dorsal skin in healthy mice after topical treatment with or without isoquercitrin on Day 5. (F) The epidermal thickness of dorsal skin in healthy mice after topical treatment with or without isoquercitrin on Day 5. All experiments were performed in six animals (*n* = 6). Data are represented as mean ± SEM

The safety of topically applied isoquercitrin on normal mouse skin was established in this study. The administration of this glycoside (20 mM) on the dorsal skin was carried out for five consecutive days to evaluate the skin tolerance. The macroscopic and microscopic observation of the en face view displayed no change of the skin surface after isoquercitrin treatment for five days as compared to the PBS control (Fig. 9A and 9B). No significant difference of the skin surface pH after isoquercitrin treatment was detected during the five days (Fig. 3C). The control group showed increased TEWL within days three to five of the PBS treatment as compared to day one (Fig. 9D). It could be due to the skin hydration by PBS. Isoquercitrin could reduce TEWL as compared to PBS control. The TEWL value of the isoquercitrin-treated group maintained a level of about 10 g/m^2^/h, indicating the preservation of the skin barrier function. The H&E staining illustrated no histological damage and immune cell infiltration after isoquercitrin delivery into the skin (Fig. 9E). No epidermal thickening was detected following the isoquercitrin intervention according to the measurement of thickness (Fig. 9F).

## Discussion

To rate the anti-AD activity of these glycosides, we first examined the cytokine/chemokine inhibition in human keratinocytes. Epidermal keratinocytes function as the predominant resources of proinflammatory mediator production in AD. IL-6, CXCL8, and CXCL1 are proinflammatory effectors that are thought to be central in AD inflammation. The Th2-dominant inflammatory chemokines RANTES and TARC play an active capacity in directing immune cells to inflammatory sites. The level of both chemokines in lesional skin correlates well with the clinical severity of AD [28]. The antagonism of these cytokines/chemokines in keratinocytes can exhibit pharmacological efficacy in AD [29, 30]. The three glycosides tested in this study were abundant in *H. cordata* Thunb. Lee et al. [31] demonstrated the usefulness of *H. cordata* Thunb extract to mitigate Th2-mediated TARC production in skin fibroblasts. We also indicated the inhibition of TARC upregulation in the stimulated keratinocytes after the treatment of these compounds. IL-6 and RANTES inhibition was observed after the treatment of isoquercitrin. Isoquercitrin and rutin, but not quercitrin, suppressed the overexpressed CXCL8. Isoquercitrin generally showed superior inhibition on cytokine/chemokine overexpression in keratinocytes.

As a recognized inflammatory mechanism, MAPKs are strongly involved in the immune disruption elicited by AD [32]. Our data manifested an inhibition of ERK phosphorylation by the three glycosides tested. The p-ERK blockade by quercetin, the aglycone form of these glycosides, has been reported previously [7]. Isoquercitrin additionally suppressed the phosphorylation of p38 in the activated keratinocytes. The other MAPK pathways such as JNK and p38 were not associated with the anti-inflammatory activity of quercitrin and rutin. Therefore, the inhibitory effect of the quercetin glycosides on the TNF-α/IFN-γ-induced phosphorylation of ERK provided the mechanistic basis for suppression of cytokines/chemokines in keratinocytes.

Monocyte-derived cells can express the markers for both macrophage and dendritic cells [33]. We employed the monocyte cell line THP-1 with differentiation as a cell model of macrophages and dendritic cells. We found that quercitrin and rutin failed to suppress the overexpression of cytokines/chemokines in the differentiated THP-1 cells. Quercitrin and rutin can restrain IL-6 and TNF-α production in lipopolysaccharide (LPS)-stimulated RAW264.7 mouse macrophages [34, 35]. This reduction could not be reproduced in our platform using our human macrophage model. Saha and Mishra [36] stated that nanoencapsulated but not free rutin can repress IL-6 and TNF-α release in THP-1 cells through the increased cell ingestion of the nanocarriers. Free rutin might show minimal uptake into THP-1 cells, leading to the insufficient ability to inhibit cell activation. As a result, in this study, isoquercitrin intervention at 10 μM in THP-1 cells significantly decreased IL-6 and RANTES but not TNF-α and CXCL8. The ethanolic extract of *H. cordata* Thunb is successful at suppressing IL-6 and nitric oxide in LPS-treated alveolar macrophages [37]. Isoquercitrin in *H. cordata* Thunb could act as a major ingredient to inhibit IL-6. Both keratinocytes and macrophages represent promising cell targets for therapeutic approaches to treat AD. Our results generally demonstrated that the glycosylated quercetins showed superior proinflammatory mediator suppression in keratinocytes than that in macrophages. Keratinocytes and macrophages can be the primary and secondary cell targets for the glycosides to mitigate inflammation in AD, respectively.

The hallmark symptom of AD is pruritus, which is associated with M2 macrophages [38]. IL-4 and IL-13 have also been implicated as sources of itching in AD [39]. Mast cells and basophils also play an essential role in the itching induced by AD [4]. T cells largely differentiate to Th2 cells and evoke IgE production in AD development. IgE mediates antigen stimulation in mast cells through binding with Fcε receptor I, resulting in degranulation and the release of histamine and β-hexosaminidase [40]. We employed the RBL-2H3 cell line as the mast cell model to examine the anti-allergic activity of the glycosides. Our data confirmed the ability of quercetin glycosides on histamine release in the sensitized RBL-2H3 cells. However, no activity was detected in the β-hexosaminidase amount. The degranulation inhibition of mast cells can be accomplished by the cell membrane stabilization [41], and quercetin glycosides might have this activity. Antihistamines are the first-line agents for relieving the itching sensation. Our result suggested that the inhibition of mast cell degranulation is another mechanism through which the glycosides improved allergic response of AD. RANTES plays a vital role in directing mast cells to inflammatory region [42]. The inhibitory potential of isoquercitrin on RANTES secretion in HaCaT and THP-1 cells inferred the synergism of pruritus amelioration by preventing histamine release and restricting mast cell infiltration.

The structural difference among the three glycosides is the glycone type. Rutin has two sugar groups, whereas quercitrin and isoquercitrin possess only one. The difference between quercitrin and isoquercitrin is an additional hydroxyl group in the 6’’-position of the sugar moiety in isoquercitrin. The cell-based study showed that isoquercitrin generally had greater anti-inflammatory activity than the others, since this glucoside-containing glycoside inhibited the concentration of more proinflammatory mediators with greater level. The 6’’-OH moiety in isoquercitrin decreases the H-donating ability through steric hindrance as compared to quercitrin, resulting in greater reactive oxygen species suppression than quercitrin [43]. Another possibility was that the σ bond between C_5’’_ and C_6’’_ can freely rotate, allowing the 6’’-OH to readily turn to the target site for reaction. The higher antioxidant activity of isoquercitrin compared to quercitrin could be the reason of greater anti-inflammatory activity in the cells tested in this study. The previous study [14] also demonstrated the greater inhibitory effect of isoquercitrin compared to quercitrin on the superoxide anion production in the stimulated human neutrophils. Further investigation is needed to elucidate the detailed mechanisms. The higher number of sugar groups in rutin compared to quercitrin and isoquercitrin led to the larger molecular size and stronger hydrophilicity. This feature of rutin was confirmed by in silico physicochemical properties. The large and hydrophilic rutinoside molecules can deteriorate the cellular uptake [44]. Rutin could be difficult to transport across the cellular membrane, contributing to the mild mitigation on cell activation.

Skin absorption at a sufficient amount is important for developing topical therapies. Quercetin has low skin permeation, which limits its application for topical therapy [14, 45]. However, the glycoside forms of quercetin could achieve efficient skin permeability and topical anti-AD therapy. We demonstrated a greater skin absorption of quercitrin and isoquercitrin compared to rutin in IVPT. The larger molecular size of rutin compared to quercitrin and isoquercitrin resulted in the decreased transport into the skin. SC is a lipophilic barrier for permeation. Rutin, with more hydrophilic characteristics than the mono-glycone glycosides, was unfavorable for diffusing across the lipophilic SC. The 3-*O*-glycosylation of quercetin significantly improves the absorption into the small intestine [46]. An investigation in rats demonstrated that orally administered isoquercitrin is absorbed better than quercetin, quercitrin, and rutin [47]. Rutin has a constrained bioavailability through the oral and cutaneous delivery [48]. Our data correlated with the previous studies. Quercitrin and rutin are facilely hydrolyzed by intestinal α-rhamnosidase and β-glycosidase to form quercetin after oral ingestion [13]. Rutin can also be hydrolyzed by α-rhamnosidase to generate isoquercitrin. No hydrolysis of the glycosides was detected in our IVPT study. This could be due to the limited amount of the glycoside hydrolase in the skin. We inferred that the quercetin glycosides could keep their anti-inflammatory activity after entering into the skin. The in silico molecular docking manifested the strongest interaction of ceramides II and VI with isoquercitrin and quercitrin, respectively. Both ceramides in the SC layer were important for the facile absorption of mono-glycone glycosides. Rutin showed the strongest interaction with free fatty acids among the glycosides tested. This suggested that free fatty acids were not important for judging the skin absorption of the glycosides.

The skin barrier defect found in AD is primarily detected in the SC [49]. The cytokines associated with Th1, Th2, and Th17 are responsible for the epidermal lipid defect in AD [50]. Reduced ceramide levels in AD lesions are a remarkable sign of increased skin barrier damage and drug absorption [51]. SC stripping and lipid removal largely increased the skin penetration of the glycosides, indicating a facile skin delivery of the glycosides into AD lesions. The skin deposition of quercitrin and isoquercitrin in the barrier-defective skin was approximate. The 6’’-OH group in the glycones did not significantly influence the skin permeation of the glycosides. A risk of the compromised skin barrier in AD is the possible delivery of topically applied drugs into systemic circulation, evoking adverse effects. The *S* value indicated a better cutaneous targeting of isoquercitrin than quercitrin in the barrier-defective skin. Isoquercitrin could be a potential candidate for treating AD because of the satisfied skin absorption and limited risk of systemic absorption.

A significant mitigation of AD-like lesions by topical isoquercitrin was found according to the skin appearance, amelioration, and proinflammatory mediator reduction. The anti-cytokine/chemokine effect of isoquercitrin on AD approximated that of commercial tacrolimus ointments. The interleukins, TNF-α, and RANTES examined in the lesional skin could be derived from different cells in vivo, including keratinocytes, T cells, macrophages, mast cells, and neutrophils. For instance, it has been shown that mast cells are one of the main cell types manifesting the secretion of interleukins, TNF-α, and RANTES in AD [52]. Our cell-based experiments demonstrated the ability of isoquercitrin on the regression of cytokine/chemokine overexpression in the activated cells. IL-4 together with IL-13 and TNF-α causes the downregulation of filaggrin, loricrin, and involucrin, which are associated with the skin barrier function, limiting the synthesis of free fatty acids in SC [51]. The inhibition of these cytokines can restore these regulators to reserve the barrier function in AD [50]. Isoquercitrin partially decreased TEWL to improve the barrier function in AD-simulated mice. This could be due to the suppression of IL-4 and IL-13 by isoquercitrin. A dysfunctional epidermal barrier elevates the local concentration of cytokines/chemokines in AD, facilitating the migration of inflammatory cells into the lesional site [2]. It was expected that topical isoquercitrin could prevent this vicious cycle. We showed that the recruitment of macrophages and neutrophils was hindered by topical isoquercitrin. Some granulocytes such as mast cells, basophils, and neutrophils accumulate in AD skin [1]. The cytokine/chemokine release from the activated keratinocytes, macrophages, and mast cells can attract neutrophils to the lesion [52, 53].

The cell viability study on keratinocytes, macrophages, and mast cells showed an acceptable cytotoxicity by isoquercitrin. The in vivo irritation test performed in this study proved the safe use of topical isoquercitrin. The reduction in skin moisture can be determined using the elevated TEWL level. Isoquercitrin even reduced the TEWL level in healthy animals, suggesting the enhanced cutaneous hydration by this compound could maintain the water content in the skin. Dry skin is usually found in AD. Emollients should be used frequently in AD patients to prevent this dryness. Besides the anti-inflammatory activity of isoquercitrin against AD, this glycoside may have emollient properties for topical use. Quercitrin and rutin have been reported to have anti-AD activity based on cell- and in vivo-based studies [54, 55]. In the present investigation, we found a superior anti-inflammatory effect of isoquercitrin compared to quercitrin and rutin. The intention of AD treatment is to mitigate inflammation, recover the barrier function, and alleviate itching. Our study verified the capability of isoquercitrin to accomplish these objectives. The oral bioavailability of quercetin and its glycosides is low [12, 50], and these compounds are also quickly cleared due to their short half-life in circulation. Topical delivery offers an advantageous route to avoid these problems. As a result, topically applied isoquercitrin was found to be beneficial for anti-AD therapy.

Some limitations in this study should be addressed. Firstly, the laboratory animals may not fully replicate the pathogenesis of human AD. It remains uncertain whether the experimental results obtained from the DNCB model and the observed facile absorption of isoquercitrin in baby pig skin can be replicated by humans. The SC and viable skin of baby pigs is thinner compared to adult human skin. The pigs usually have more densely packed hair follicles and larger number of sebaceous glands as compared to human skin [56]. Although the baby pig skin and human skin share structural similarity in terms of overall organization, lipid profile, and collagen content, it should be cautious to extrapolate the permeation data of pig skin into human condition. The optimization of applied dose, therapeutic duration, and dosage forms is also demanded to maximize the efficacy. Future studies must validate the accuracy of this study’s findings to address these limitations effectively.

## Conclusions

The present work validated that glycosylated quercetins in the 3-OH site suppressed the stimulation of the keratinocyte, macrophage, and mast cell models involved in AD pathogenesis. These compounds could arrest the upregulation of proinflammatory effectors in the activated keratinocytes via ERK signaling. Histamine release from the RBL-2H3 cells decreased after treatment with the glycosides, supposing the potential in alleviating pruritus in AD. Isoquercitrin generally showed greater anti-inflammatory activity compared to the other glycosides in the cell-based study. The IVPT exhibited higher skin permeation of mono-glycone compounds than the glycoside with dual sugar moieties. Topical isoquercitrin prevented the development of AD-like inflammation in DNCB-sensitized mice. The topical delivery of isoquercitrin improved the lesions through the inhibition of cytokines/chemokines, epidermal hyperplasia, and immune cell accumulation. Our findings identified isoquercitrin as a candidate for alleviating inflammation in AD.

## Data Availability

No datasets were generated or analysed during the current study.
